# Comparative Analysis of Acral Melanoma in Chinese and Caucasian Patients

**DOI:** 10.1155/2020/5169051

**Published:** 2020-10-06

**Authors:** Kai Huang, Yu Xu, Emmanuel M. Gabriel, Subhasis Misra, Yong Chen, Sanjay P. Bagaria

**Affiliations:** ^1^Department of General Surgery, Brandon Regional Hospital, HCA Healthcare/USF Morsani College of Medicine, Brandon, FL, USA; ^2^Department of Musculoskeletal Oncology, Fudan University Shanghai Cancer Center, Shanghai, China; ^3^Department of Oncology, Fudan University Shanghai Medical College, Shanghai, China; ^4^Department of General Surgery, Mayo Clinic Florida, Jacksonville, FL, USA

## Abstract

**Background:**

Acral melanoma (AM) is a rare subtype of melanoma, which is one of the least common in Caucasian patients but is a common subtype of melanoma in Chinese patients. It is unclear if prognosis differs between Chinese and Caucasian patients diagnosed with AM. The aim of our study is to investigate patient characteristics and survival differences between Chinese and Caucasian AM patients.

**Methods:**

Two large institutional melanoma databases from Fudan University Shanghai Cancer Center (FUSCC) and Mayo Clinic enterprise were retrospectively reviewed from 2009 to 2015. Clinicopathologic and survival data were collected and analyzed between the two groups. The primary outcome was disease-specific survival (DSS) and was calculated using the Kaplan Meier (KM) method.

**Results:**

The Chinese group presented with more advanced disease compared with Caucasians: thicker Breslow depth (median 3.0 mm vs. 1.2 mm, *p*=0.003), more ulcerated disease (66.1% vs. 29%; *p* < 0.001), and advanced stages (stage II/III 84.3% vs. 37.1%; *p* < 0.001). No significant difference was identified in terms of age at diagnosis, location, histologic subtypes, or node positive rate. The 5-year DSS rate was 68.4% and 73% (*p*=0.56) in Chinese and Caucasians AM patients, respectively. Male gender, Breslow thickness, ulceration, and positive sentinel lymph nodes were independent poor prognostic factors on multivariate analysis.

**Conclusions:**

There appears to be no difference in stage-stratified survival between Chinese and Caucasians, supporting the implementation of clinical trials for AM that could include both Chinese and Caucasian patients.

## 1. Introduction

Malignant melanoma is the most common potentially fatal neoplasm of the skin, and its incidence has increased steadily over the last several decades [1]. Based on the anatomic location of the tumor and the degree of UV exposure, melanoma can be classified into four subtypes: (1) melanomas that occur on the skin without chronic sun-induced damage (non-CSD); (2) melanomas on the skin with chronic sun-induced damage (CSD); (3) mucosal melanomas; and (4) acral melanomas [2]. AM occurs in areas with little to no sun exposure, such as palms, soles, or nail apparatus. It is the least common subtype of cutaneous melanoma in Caucasians (1–7%), but it is the most common type of melanoma diagnosed in nonwhite populations (Asians, Hispanics, Black American, etc.), [3–6] accounting for 58% of all cutaneous melanomas in Asians. With rare occurrence, its lower awareness and unusual presentation delay in diagnosis, the diagnosis of AM is often delayed in minorities, leading to more advanced stages and worse prognosis than other subtypes of melanoma.

It has been suggested that racial differences exist in cutaneous melanoma [7]. However, results in the literature are still not consistent. Reintgen et al. [8] reported differences in stage-specific melanoma outcomes between Blacks and Whites. However, Hemmings et al. [9] reported no differences in outcomes in non-Whites versus Whites who were stratified by stage at initial diagnosis. Racial difference in AM has not been investigated between Asians and Caucasians.

Due to the low incidence of melanoma in China, only a few case series of Asian AM have been published. The clinical and pathological characteristics, treatment, and prognosis of Chinese AM patients have never been compared with Caucasian patients. We presented two cohorts of AM patients following curative resection and SLNB in two large referring centers in China and the US, aiming to investigate characteristics and survival differences between Chinese and Caucasian AM patients.

## 2. Patients and Methods

### 2.1. Patient Selection

A consecutive series of AM patients who underwent surgical treatment at FUSCC and Mayo Clinic Enterprise between December 1, 2009, and December 1, 2015, were retrospectively selected from two institutional databases. Chinese or Caucasian patients presented with primary melanoma, with a tumor located at palmar, plantar, or subungual area, clinical node negative disease, treated initially with surgical excision were included in the study. Patients with one or more invasive tumors, melanomas located in nonacral locations, clinically positive regional lymph nodes, stage IV disease, or recurrent disease were excluded from our study. Primary tumor and sentinel lymph node specimens were reviewed and confirmed by experienced dermatopathologists at each institution. 280 Chinese AM patients at FUSCC and 62 Caucasian AM patients at Mayo clinic Enterprise were included in our study. This study was approved by the Institutional Review Board (IRB) at both institutions.

Patients were staged according to the American Joint Committee on Cancer (AJCC) 7^th^ Edition Staging System. Demographic and clinicopathological data included age at diagnosis, gender, tumor location, greatest dimension, pathological stage, histological type, Breslow depth, Clark level, ulceration, Sentinel Lymph node (SLN) status, surgical procedure, and adjuvant treatment. The great dimension of tumor was measured clinically according to the clinical documentation. Disease status at the latest contact was categorized as NED (alive with no evidence of disease), AWD (alive with disease), DFD (death from disease), DFO (death from other causes), and DUK (death and disease status unknown). DSS was defined as the time from pathologic diagnosis to the time of death due to melanoma or last follow-up. Patients' current disease status at the end of follow-up and their first recurrence were listed as well, which are classified as node-only (regional), local only, in-transit disease, or distant organ metastasis (liver, lung, brain, bone, etc.).

### 2.2. Surgical Procedures, SLNB

Wide local excision of the tumor was performed with an adequate margin according to National Comprehensive Cancer Network (NCCN) guidelines. Amputation was applied for subungual lesion if the adjacent joint was involved in the adequate resection margin. Reconstruction of the primary defect was performed by either skin graft, or second intention healing at non-weight-bearing area, or rotational flap on weight-bearing area at the discretion of the primary surgeon or plastic surgeon. Lymphatic mapping techniques, including radioisotope, methylene blue, or both, were used when performing SLNB.

SLNB was performed for all patients with nonpalpable lymph nodes, and SLN status was evaluated by the frozen section intraoperatively at FUSCC. For those with node positive disease intraoperatively, complete lymph node dissection was performed subsequently, as well as those with node positive disease on final pathology. At Mayo, SLNB was performed for patients with Breslow thickness >1 mm, and SLN status was evaluated only on final pathology. Patients with sentinel lymph node positive disease underwent completion of lymph node dissection or close observation with ultrasound at the discretion of primary surgeons.

### 2.3. Adjuvant Therapy

At FUSCC, adjuvant therapies included immunotherapy alone or immunotherapy combined with chemotherapy. Immunotherapy included Interleukin-2 (IL-2) or interferon (IFN)-alpha. The chemotherapy regimen included Dacabazine plus Cisplatin, or Temozolomide alone. At Mayo, adjuvant therapy included immunotherapy with IL-2 alone.

### 2.4. Statistical Analysis

The primary outcome was the five-year DSS. The KM method was used to develop the survival curves and estimate DSS. Categorical variables were compared between the two groups using the Chi-square test, and continuous variables were compared using analysis of variance (ANOVA). Univariate and multivariate Cox proportional hazard models were applied to identify factors that are associated with OS, and HRs and 95% CIs were reported. Variables that were significant in univariate analysis were included in multivariable Cox proportional hazard analysis. All statistical analysis was performed using IBM SPSS v23.0. Statistical significance was defined as *p* < 0.05.

## 3. Results

### 3.1. Patient Demographics and Characteristics

The clinical and pathological features of the two groups were summarized in Table 1. No significant difference in age at diagnosis between Chinese and Caucasians (median 60.5, vs. 64.5, *p*=0.25). AM was more common in males in both groups, with a male to female ratio of 1.22 : 1 in Chinese patients compared to 1.14 : 1 in Caucasians (*p*=0.8). Tumor size was larger in Chinese AM than in Caucasians (median 2.50 cm vs. 1.0 cm, *p* < 0.001). Chinese patients had more advanced disease than Caucasians. Chinese AM had thicker tumor (Breslow thickness, median 3.0 mm, vs. 1.0 mm, *p*=0.003), more ulcerated disease (66.1% vs. 29%, *p* < 0.001), and more stage II-III disease (84.3% vs. 37.1%, *p* < 0.001) as compared to Caucasians. Volar sites (lower limb) were more frequently involved than subungual melanoma in both groups (Chinese 68.6% vs. 31.4%, Caucasian 61.3% vs. 38.7%). Acral lentiginous melanoma was the predominant subtype of AM in both groups (92.8% Chinese vs. 96.8% Caucasian, *p*=0.21).

### 3.2. Treatments

#### 3.2.1. SLNB and CLND

All patients in the Chinese group had SLNB, 30.4% of whom had positive SLN. Fifty-four percent of patients in the Caucasian group received SLNB, 35.3% of whom had positive SLN. The mean number of SLN was 3.07 in Chinese patients compared to 2.0 in Caucasian patients. Among SLN positive patients, 74/85 (87%) of patients underwent completion lymph node dissection (CLND) in the Chinese group, with a mean of 8.7 (1–22) LNs removed and 27/77 (35.1%) of patients had positive nonsentinel nodes. In the Caucasian group, 8/12 (66.7%) underwent CLND, 23.1 (11–48) LNs were removed, and 2/8 (25%) of patients had positive nonsentinel nodes.

#### 3.2.2. Surgery and Adjuvant Therapy

The majority of patients with volar tumors (Chinese 88.5% vs. Caucasian 73.7%) underwent wide local excision (WLE), whereas amputation was performed more often for subungual tumors (Chinese 93.2% vs. Caucasian 79.2%). The majority of Chinese patients (84.7%) underwent adjuvant therapy, among which 97.5% received immunotherapy (IL-2 or IFN-alpha alone or combined) and 2.5% received immunotherapy combined with chemotherapy. In the Caucasian group, only 6.5% of patients received immunotherapy (IL-2). Regarding adjuvant therapy by stage, 45% (37/81) stage 0/I patients, 79.5% (136/171) stage II patients, and 77.5% (69/89) stage III patients underwent adjuvant therapy.

#### 3.2.3. Prognostic Factors and Survival Analysis

The median follow-up was 43 months (3–101 m) in the Chinese group and 24.5 months (2–75 m) in the Caucasian group. 25.7% (72/280) Chinese patients died from melanoma, whereas 11.3% of (7/62) Caucasian patients died of the disease. 115/280 (41.1%) recurrences occurred in the Chinese group, with regional node recurrence being the most common (33%), followed by distant organ metastasis (32.2%), in-transit and local recurrence. The Caucasian group had 27.4% (17/62) recurrences, with in-transit metastasis being the most common (35.2%), followed by local recurrence (29.4%), distant organ (23.5%), and nodal recurrence (11.4%) (Table 2).

Gender, pathological stage, Breslow thickness, Clark level, ulceration, and SLN status were associated with DSS on univariate analysis. The race was not a prognostic factor and the hazard ratio was 1.26 (0.58–2.76) in Chinese compared to Caucasian AM patients. Gender, Breslow thickness, presence of ulceration, and positive SLN were independently prognostic factors on multivariate analysis (Table 3). Positive SLN was associated with poor DSS with a HR 4.10, (95%CI 1.54–10.92, *p*=0.003). In the subgroup analysis of AM patients who underwent SLNB (*n* = 315), patients who had a positive SLN had a 5-year DSS of 44.0% (95% CI: 25.9–55.3%) compared to a 5-year DSS of 76.2% (95% CI: 67.4–85.0%) in SLN negative patients (*p* < 0.001).

**Figure 1:**
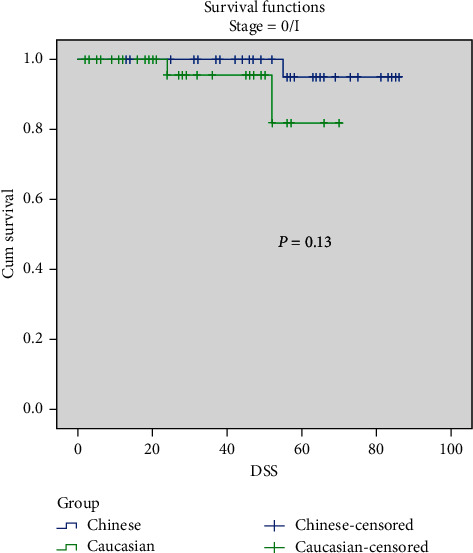
Stage-stratified 5-yr DSS in Chinese and Caucasian groups. Stage 0/I, *p*=0.13.

**Figure 2:**
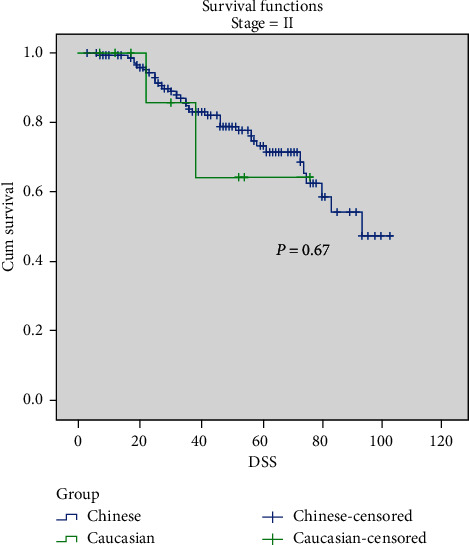
Stage-stratified 5-yr DSS in Chinese and Caucasian groups. Stage II, *p*=0.67.

**Figure 3:**
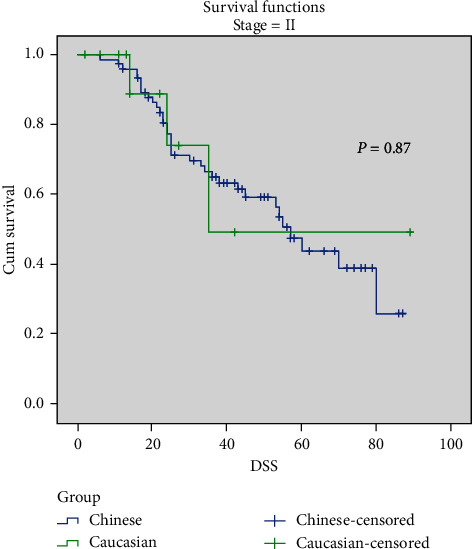
Stage-stratified 5-yr DSS in Chinese and Caucasian groups. Stage III, *p*=0.87.

Comparing Chinese and Caucasians, the overall 5-year DSS rate was 68.4% vs. 73% (*p*=0.56), respectively. No significant difference was found in stage-stratified DSS. Chinese had a 5-year DSS rate of 43.9% compared to 49.4% in Caucasian patients in Stage III disease, 73.3% vs. 64.3% in Stage II disease, and 95.0% vs. 94.4% in Stage I disease (Figures 1–3). There was no DSS significance between Chinese and Caucasian patients when controlled for Breslow thickness as well (data not shown).

## 4. Discussion

AM is a distinct subgroup of cutaneous melanoma occurring on the palmoplantar and subungual sites with specific histological and clinicopathological features, regardless of histologic type (if acral lentiginous or not) [10]. It is the most commonly occurring subtype of melanoma in Asian populations and is known to have a worse prognosis than nonacral melanoma [7, 11], likely due to its late presentation and diagnosis or its intrinsic high aggressiveness [12]. Racial differences have been investigated in a few case series [11, 13] and a population-based analysis on cutaneous melanoma [7]. However, it is still controversial. Black Americans were reported to have lower DSS compared to whites and other races [13]. Due to its rarity, no study has compared characteristics and outcomes in AM between Chinese and Caucasian patients. Our study represents the first direct comparative study of a large series of Chinese and Caucasian AM patients from two tertiary referral institutions. To capture the disease process for newly diagnosed AM treated in a standard fashion, patients were selected from a consecutive time period, with surgery being the initial treatment. Patients presenting with recurrent disease or metastatic disease were excluded.

Considering clinical and pathological characteristics, Chinese and Caucasian patients were very similar in terms of mean age of diagnosis (the early 1960s), no significant gender predominance, volar (lower limb) predilection, and acral lentiginous type predominance, which were consistent with prior studies [14]. This study also found that Chinese patients presented with advanced disease compared to Caucasian patients. This is consistent with other studies that have reported a higher percentage of T4 disease in Asia/Pacific Islanders compared with white skin and black skin patients [7], as well as a high proportion of Breslow T4 disease in Chinese and Koreans (40.8% and 33%, respectively). Lv et al. also reported a mean Breslow thickness of 4.9 mm and 47.9% of ulceration in their series [10, 15]. Delay in diagnosis of AM with a duration ranging from 1 to 3.7 years was described in the literature [15, 16], due to hidden site, frequent lack of pigmentation, and lack of recognition, and misdiagnosis by dermatologists was an explanation of the advanced stage disease in Chinese [15].

Decreased DSS was associated with male gender, thick Breslow depth, high Clark level, presence of ulceration, advanced pathological stage, and positive SLN. Gender has been reported to be an independent prognostic factor [11, 17, 18]. We also found that male sex was associated with a worse prognosis, even after controlling for other factors. In the Chinese group, male patients had a 5-year DSS of 59.4% compared with 77.9% in female patients, whereas, DSS was 55.1% in males versus 93.8% in females in the Caucasian group. Breslow thickness and presence of ulceration have been shown to be important prognostic factors for cutaneous melanoma and AM [7, 11, 19]. SLN status has proven to be an important prognostic factor for cutaneous melanoma 26–30 and has been reported to be an important prognostic factor for AM as well [19]. The positive rate of SLN was 40% (63/157) AM patients who underwent SLN biopsy in Bello et al.'s study [19] and was 30.4% in the Chinese group and 35% in the Caucasian group in our study. SLN status (HR 4.10, 1.54–10.92, *p*=0.003) was the strongest prognostic factor in AM patients who underwent SLN biopsy. The SLN positive rate was 2.9% in T1, 20.9% in T2, 39.2% in T3, and 48.7% in T4 disease. Five-year DSS was 44.0% in patients with positive SLN compared with 76.2% in patients with a negative SLN, which is consistent with prior literature.

The prognostic value of racial difference has been investigated in the literature, but its implication on outcomes remains unclear. Some studies have identified race as an independent prognostic factor, whereas others have shown similar survival rates among different racial groups after controlling for stage [3, 7, 14]. Bradford et al. reported on a large population of Acral Lentiginous Melanoma patients and found the 5-year DSS rates were highest in Non-Hispanic whites (82.6%), intermediate in black Americans (77.2%), and lowest in Asian/Pacific Islanders (70.2%) [7]. However, no survival difference was found between Caucasians and Asian/Pacific Islanders, after adjusting for stage and thickness. Lv et al. reported on a large series of Chinese AM patients and found the 5-year DSS to be 53.5%, [15] which is worse than reported in Caucasians (e.g., the 5-year DSS of 70% reported by Bello et al. [19], 71% by Kuchelmeister et al. [20], and 76% by Phan et al. [21]). In our study, race was not a prognostic factor (HR 1.26, 95% CI 0.59–2.76; *p*=0.56). Chinese patients had a more advanced disease as compared to Caucasian patients. However, there was no significant difference in the 5-year DSS survival between the two groups (68.4% vs. 73%, *p*=0.56) after adjusting for stage and thickness, which was consistent with the literature. In our cohort, a much higher percentage of Chinese patients received adjuvant therapy (84.7% vs. 6.5%), which may impact the DSS of Chinese patients. One hypothesis is that tumor biology or genetic alterations in AM between the two groups has a similarity that plays a role. High focal amplifications, including CCND1, CDK4, and GAB2, and low mutation rates on BRAF, NRAS, and KIT have been reported in Caucasians [2, 22]. A recent study of genetic alterations of Chinese showed CDK4 gain (39.5%), CCND1 gain (26.7%), and P16^INK4a^ loss (60.3%) [23]. Also, positive SLN, which was the most important detrimental prognostic factor, was higher in the Caucasian group (35.3% vs. 30.4% in Chinese), though not statistically significant. It may be an explanation for the survival difference between the two groups.

Evidence suggested that adjuvant systemic therapy especially new agents such as CTLA-4 blockade, an immune checkpoint inhibitor, has a sustained positive impact on DSS [24, 25]. Adjuvant IFN alfa, particularly high-dose IFN alfa, has been widely used in patients with melanoma for many years. Its results on survival still vary across clinical trials [26, 27]. In our study, patients with adjuvant therapy were not associated with improved DSS. During our study period, immunotherapy was consisted of an old regimen including interferon or interleukin-2, as opposed to the more novel and effective agents that utilize immune checkpoint blockade. The expected effect of immunotherapy within our study would be lower than that of the newer anti-CTLA4 and anti-PD1 agents involved in immune checkpoint blockade. The indication of adjuvant therapy in our patients was not standardized as the fact that a high percentage of patients with stage I and stage II underwent adjuvant therapy. Also, a low percentage of stage III patients who will benefit more from adjuvant therapy enrolled in the study may be another reason that adjuvant therapy did not have a positive impact on DSS.

The major strength of this study is that it includes the largest number of primary AM patients. All of our Chinese patients had sentinel lymph node biopsy, allowing more accurate pathological staging. We acknowledge that there are limitations to our study. Margin status and mitotic rates of the primary tumor were not recorded in the FUSCC database and could not be analyzed, both of which could be potential prognostic factors. Nonsentinel lymph nodes status was not recorded in our database, which could be associated with high regional node recurrence. The median follow-up period was nearly four years in the Chinese group versus two years in the Caucasian group. A shorter follow-up interval and limited patients included in the Caucasian group could lead to statistical limitations in identifying survival differences between the two cohorts.

In conclusion, this study represents the first (and one of the largest) contemporary series investigating stage 0–III AM in Chinese and Caucasians, from two large referral centers. Our results showed there appears to be no difference in survival between Chinese and Caucasians diagnosed with AM, after controlling for stage and thickness, even though the Chinese patients presented with more advanced disease. Our results imply that the biological course of AM is likely similar between Chinese and Caucasian patients. Future studies are warranted to clarify and expand on the biological differences of AM between different racial groups. As Asian countries have a high incidence of AM, our data support the implementation of clinical trials of stage 0–III AM that could include both Chinese and Caucasian cohorts.

## Figures and Tables

**Table 1:** Clinical and pathological parameters of AM patients in Chinese (*n* = 280) and Caucasians (*n* = 62).

Parameter	Chinese (*n* = 280) (%)	Caucasian (*n* = 62) (%)	*p* value
Age at diagnosis (years)			0.25
Median (IQR)	60.5 (51–70)	64.5 (49.5–75.3)	

*Gender*			0.80
Male	154 (55)	33 (53.2)	
Female	126 (46)	29 (46.8)	
Male to female ratio	1.22 : 1	1.13 : 1	

*Pathologic stage*			<0.001
0/I	43 (15.3)	38 (61.2)	
II	160 (57.1)	11 (17.7)	
III	76 (27.2)	12 (19.4)	
Unknown	1 (0.4)	1 (1.6)	

*Largest Diameter (cm)*	(0.4–10.0)	(0.2–4.5)	<0.001
Median (IQR)	2.5 (1.7–3.5)	1.0 (0.43–2.0)	
Unknown	90 (32.0)	18 (29.0)	

*Location*			0.27
Volar	192 (68.6)	38 (61.3)	
Foot	191 (68.2)	37 (59.6)	
Hand	1 (0.4)	1 (0.7)	
Subungual	88 (31.4)	24 (38.7)	

*Histology*			0.21
Acral lentiginous	260 (92.8)	60 (96.8)	
Nodular	8 (2.9)	1 (1.6)	
Superficial spreading	12 (4.3)	1 (1.6)	

*Breslow thickness (mm)*			0.003
Median (IQR)	3.0 (1.7–5.0)	1.2 (0.65–2.8)	
Unknown	54 (19.3)	11 (17.7)	

*Clark Level*			<0.001
I	10 (3.6)	8 (12.9)	
II/III	28 (10.0)	22 (35.5)	
IV/V	164 (58.6)	18 (29.0)	
Unreported/unknown	78 (27.8)	14 (22.6)	

*Ulceration*			<0.001
Yes	185 (66.1)	18 (29.0)	
No	87 (31.1)	44 (71.0)	
Unreported/unknown	8 (2.8)	-	

*SLN biopsy*			N/A
Yes	280 (100)	34 (54.8)	
No	—	28 (45.2)	

*SLN biopsy positive*			0.56
Yes	85 (30.4)	12 (35.3)	
No	195 (69.6)	22 (64.7)	

Number of SLN resected			
Mean (SD)	3.1 (2.05)	2.0 (1.39)	
CLND after SLNB+	74/85 (87)	8/12 (66.7)	
Number of total LNs resected, mean (SD)	8.7 (4.35)	23.4 (12.1)	
CLND positive	27/77 (35.1)	2/8 (25)	0.44

*Surgery*			N/A
WLE	173 (61.8)	30 (48.4)	
Amputation	88 (31.4)	27 (43.5)	
Mohs surgery	—	3 (4.8)	
Unknown	19 (6.8)	2 (3.2)	

*Adjuvant therapy*			N/A
Yes	238 (84.7)	4 (6.5)	
No	32 (12.4)	58 (93.5)	
Unreported/unknown	10 (3.9)	—	

**Table 2:** Patient disease status and recurrence of AM patients in Chinese and Caucasians.

Parameter	Acral melanoma in Chinese	Acral melanoma in caucasian
*Patient disease status (%)*		
NED	174 (62.1)	48 (77.4)
DFD	67 (23.9)	5 (8.1)
AWD	30 (10.7)	8 (12.9)
DFO/DUK	9 (3.3)	1 (1.6)

*First Recurrence (%)*	*N* = 115 (41.7)	*N* = 17 (27.5)
Local	7 (6.1)	5 (29.4)
Regional LN	38 (33.0)	2 (11.8)
In transit	18 (15.7)	6 (35.2)
Distant organ mets (lung, liver, brain, bone, etc.)	37 (32.2)	4 (23.5)
Unknown	15 (13.0)	—

Disease status at the latest contact: NED (alive with no evidence of disease), AWD (alive with disease), DFD (death from disease), DFO (death from other causes), and DUK (death and disease status unknown).

**Table 3:** Prognostic factors associated with DSS in AM patients.

Parameter	Univariate analysis		Multivariate analysis	
	Hazard ratio (95% CI)	*p* value	Hazard ratio (95% CI)	*p* value
*Age (years)*		0.08		
≤60 (*N* = 166)	0.68 (0.43–1.05)			
>60 (*N* = 176)	1.0			

*Gender*		0.001		0.011
Male (*N* = 187)	2.21 (1.38–3.55)		1.99 (1.17–3.38)	
Female (*N* = 155)	1.0		1.0	

*Chinese vs. Caucasian*		0.56		
Chinese (*n* = 280)	1.26 (0.58–2.76)			
Caucasian (*n* = 62)	1.0			

*Pathologic stage*		<0.001		
0/I (*n* = 81)	0.08 (0.03–0.27)			
II (*n* = 171)	0.42 (0.27–0.66)			
III (*n* = 89)	1.0			

*Location*		0.69		
Volar (*n* = 230)	0.91 (0.57–1.46)			
Subungual (*n* = 112)	1.0			

Breslow thickness (mm)	1.06 (1.03–1.09)	<0.001	1.05 (1.01–1.09)	0.018

*Clark Level (%)*		<0.001		
Unknown (*n* = 91)	0.42 (0.23–0.77)			
I/II/III (*n* = 68)	0.26 (0.11–0.60)			
IV/V (*n* = 183)	1.0			

*Histology*		0.93		
Nodular (*n* = 9)	1.01 (0.25–4.12)			
Superficial spreading (*n* = 12)	0.81 (0.25–2.57)			
Acral lentiginous (*n* = 321)	1.0			

*Ulceration*				
Yes (*n* = 203)	4.06 (2.20–6.57)	<0.001	3.39 (1.20–8.23)	0.026
No (*n* = 130)	1.0		1.0	

*SLN biopsy status*		<0.001		0.003
Positive (*n* = 97)	3.45 (2.19–5.45)		4.10 (1.54–10.92)	
Negative (*n* = 217)	1.0		1.0	

*Surgery*		0.88		
Amputation (*n* = 115)	1.04 (0.64–1.68)			
WLE (*n* = 203)	1.0			

*Adjuvant therapy*		0.60		
Yes (*n* = 242)	0.87 (0.51–1.47)			
No (*n* = 90)	1.0			

## Data Availability

The data that support the findings of this study are available from the corresponding author upon request. The data are not publicly available due to privacy or ethical restrictions.
